# Sensing of Quercetin With Cobalt-Doped Manganese Nanosystems by Electrochemical Method

**DOI:** 10.7759/cureus.56665

**Published:** 2024-03-21

**Authors:** Sree Thalir, Sherin Celshia Susai, Muthamizh Selvamani, Vasugi Suresh, Sathya Sethuraman, Karthikeyan Ramalingam

**Affiliations:** 1 Physiology, Saveetha Dental College and Hospitals, Saveetha Institute of Medical and Technical Sciences, Saveetha University, Chennai, IND; 2 Medical Physiology, Saveetha Dental College and Hospitals, Saveetha Institute of Medical and Technical Sciences, Saveetha University, Chennai, IND; 3 Oral Pathology and Microbiology, Saveetha Dental College and Hospitals, Saveetha Institute of Medical and Technical Sciences, Saveetha University, Chennai, IND

**Keywords:** biosensor, biosensor-based detection, nanotechnology biosensors, xray diffraction, scanning electron microscopy, quercetin, nanoparticles, nanospheres, cobalt doped, manganese oxide

## Abstract

Background: The pressing need for precise, quick, and affordable detection of diverse biomolecules has led to notable developments in the realm of biosensors. Quercetin, a biomolecule rich in flavonoids predominantly found in our diet, is sensed by the electrochemical method. The electrochemical properties show remarkable improvement when Mn_2_O_3_ (MO) is doped with cobalt (Co).

Aim: This study aimed to investigate the biomolecule sensing of quercetin using Co-doped MO by electrochemical method.

Materials and methods: Co-doped MO nanospheres were prepared by hydrothermal method. The crystal structure of the synthesized material was evaluated by using X-ray diffraction analysis. The sample morphology was assessed by using field emission scanning electron microscopy (FE-SEM) techniques. The cyclic voltammetry technique was used for the detection of quercetin biomolecules.

Results: The synthesized Co-doped MO appeared to be spherical in morphology in FE-SEM. Energy-dispersive X-ray spectroscopy showed the only presence of Co, Mn, and O, which confirmed the purity of the sample. The modified electrode sensed the biomolecule with a higher current of 7.35 µA than the bare glassy carbon electrode of 6.1 µA.

Conclusion: The Co-doped MO exhibited enhanced conductivity, reactivity, and electrochemical performance. This tailored approach will help in the optimization of material properties toward specific biomolecule sensing applications.

## Introduction

Biosensors are tools that react to specific analytes in the given sample. They use electrochemical, optical, or other transducers in conjunction with a biological recognition system to transform the analyte's concentration into an electrical signal. These kinds of tools are used in the bioprocessing, medical diagnostics, agricultural, and environmental monitoring sectors [1]. In recent years, the field of biosensors has undergone remarkable advancements, due to the urgent need for accurate, rapid, and cost-effective detection of various biomolecules. The advantage of biosensors is their potential to identify molecules more quickly than conventional approaches [2]. Among the various sensing strategies, electrochemical biosensing has emerged as a promising avenue because of its exceptional sensitivity, selectivity, and compatibility with miniaturization for point-of-care applications [3]. Currently available biosensors are classified as optical, thermal, mass, or electrochemical sensors depending on the form of the transducer [4]. 

The transition metal oxide nanostructures have been utilized as sensing platforms, with cobalt-doped Manganese oxide (Co-Mn_2_O_3_) being an increasingly investigated candidate [5]. MO possesses inherent properties, including high surface area, tunable morphology, and facile synthesis, which make it a compelling candidate for constructing sensing interfaces [6]. However, its electrochemical performance, especially in terms of sensitivity and stability, can be significantly enhanced through strategic doping with transition metal ions. As a versatile dopant, Co offers opportunities to tailor the electronic and structural properties of Mn_2_O_3_ (MO), thereby influencing its electrochemical behavior as a biomolecule sensor [7].

In the realm of biosensing, the detection of biomolecules such as proteins, nucleic acids, enzymes, and pathogens holds paramount significance [8]. Electrochemical techniques offer a direct and label-free means of probing these bio-analytes, relying on the inherent electrochemical activity of redox-active species utilizing bio-recognition events to induce detectable changes in the electrochemical signal. The integration of Co-MO nanostructures into these platforms introduces a multifaceted approach to enhance the sensitivity and selectivity of biomolecule detection [9]. The intrinsic reactivity of MO and the redox characteristics of Co can boost the selectivity for target biomolecules, improve the kinetics of electron transfer, and increase stability [10,11]. 

Quercetin, a naturally occurring flavonoid derived from quercetum, or oak forest has been used. It is named after Quercus. It is present naturally in a wide variety of plants, including fruits like berries, grapes, and apples, vegetables like onions, spring onions, tea, and tomatoes, and many other plant parts [12,13]. Quercetin's antioxidant, antibacterial antidiabetic, anti-inflammatory, anti-arthritic, anti-Alzheimer's, cardiovascular, and wound-healing qualities have been reported. Its anticancer efficaciousness against different cancer cell lines has been reported more recently [14].

The methods most frequently employed to determine quercetin are capillary electrophoresis [15], liquid chromatography [16], cyclic voltammetric method [17], and electrochemical method [18]. Co-doped MO represents a promising avenue for advancing the field of biomolecule sensing through electrochemical methods. The unique combination of Co's electronic properties and MO's inherent catalytic activity offers a platform for creating highly efficient and selective sensors for a range of biomolecules.

This article aimed to check the effectiveness of Co-doped MO nanospheres on the detection of Quercetin using an electrochemical method.

## Materials and methods

Preparation of Co-doped MO

For the hydrothermal production of Co-doped MO nanocomposites, urea, Co(NO_3_)_2_, and KMnO_4_ were used as precursor materials without any further purification. Solution A was prepared by taking an equal ratio of urea and KMnO_4_ (0.2 M) and mixing it with 50 mL of double-distilled water under constant magnetic stirring. Solution B was prepared by dissolving 0.2 M percentage of Co(NO_3_)_2_ (10 mol%) in 50 mL of distilled water under magnetic stirring. Both solutions A and B were mixed by slow addition method, and vigorous stirring was done at room temperature until a homogeneous solution was created. The final reaction mixture was transferred into a 200 mL autoclave and heated to 140°C for 6 hours. The autoclave was then allowed to cool naturally to ambient temperature. The product was collected after synthesis and the reaction impurities were eliminated by repeatedly washing it with ethanol and distilled water. It was then dried at 80°C in the hot air oven for 12 hours.

Analysis of Co-doped MO 

X-ray diffraction (XRD) analysis was performed to analyze the crystalline structure of the prepared Co-doped MO. D8 advance powder X-ray diffractometer (BRUKER AXS Inc, Madison, USA) was utilized. Field emission scanning electron microscopy (FE-SEM) analysis was performed to analyze the surface morphology and microstructure of the prepared nanostructures. It was done using the JSM-IT800 Schottky field emission scanning electron microscope with energy-dispersive spectroscopy (EDS) for elemental analysis and electron backscatter diffraction (EBSD) for crystallographic analysis (JEOL Ltd, Tokyo, Japan). Energy-dispersive X-ray spectroscopy (EDAX) was performed to identify the elements present in the sample to confirm the purity of synthesized Co-doped MO.

Modification of working electrode

Before the bare glassy carbon electrode (GCE) modification, the working electrode was mechanically polished using alumina pastes with sizes of 1 μm, 0.3 μm, and 0.05 μm to achieve a mirror-like quality. It was then ultrasonically treated for a short while in double-distilled water to clean the GCE surface. After 20 minutes of ultrasonic agitation, 5 mg of Co-doped MO was dispersed in 10 mL of ethanol to create the MO_ _suspension. Ten microliters of the suspension was then drop-coated onto the GCE using the drop-coating technique, and it was allowed to dry in the air.

Cyclic voltammetry

Cyclic voltammetry (CV) is an important and widely used electroanalytical method for obtaining qualitative information about electrochemical reactions. The CV provides information on the thermodynamics of redox processes, heterogeneous electron transfer reactions, mass transfer processes, and coupled chemical reactions or adsorption processes. In CV, the potential of a working electrode is changed linearly with time starting from a potential where no redox reaction of analyte occurs. After the potential has swept the region in which one or more chemical reactions take place, the direction of the linear sweep is reversed. The cyclic voltammetric reactions of bare GCE and Co-doped MO to quercetin were performed along with the estimation of the pH effect on the detection of quercetin from 3 to 8 at 50 mV/S. All the electrochemical tests were performed using CH 1103A Electrochemical Workstation (CH Instruments Inc, Austin, TX, USA).

## Results

**Figure 1:**
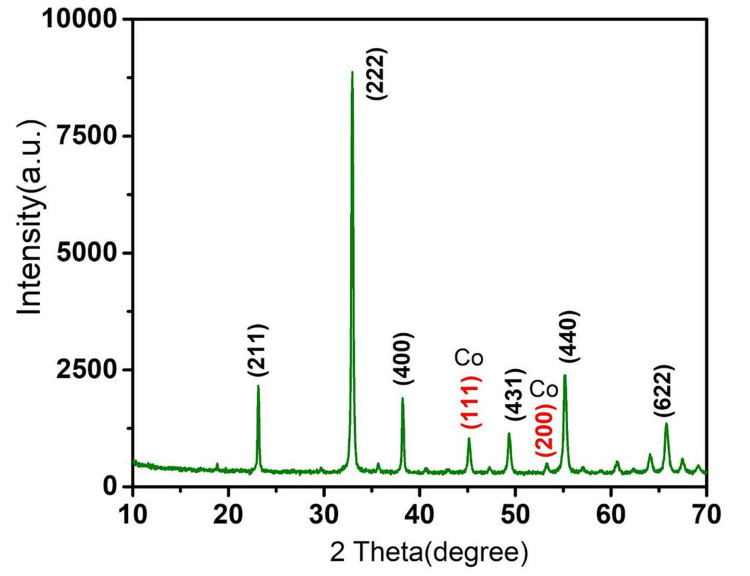
X-ray diffraction analysis of cobalt-doped manganese oxide nanostructures.

The XRD analysis shows that the Co-doped MO is crystalline in structure and it matched with JCPDs no. 41-1442. Using powder XRD, the products' crystal structures were investigated. The bixbyite crystal phase a-MO (JCPDS no. 41-1442) exhibits many peaks in all samples, including MO and Co-doped MnO. These peaks correspond to the MO (211), (222), (400), and (440) planes (Figure 1).

The absence of any further peaks originating from Co or manganese carbonate oxides was observed, indicating that the Co-doped MO sample had formed homogeneous oxide-solid solutions. Furthermore, when comparing the Co-doped samples to the pure MO, a noticeable drop in peak intensity was seen, indicating that the doping had a negative impact on the crystallization (Figure 1).

**Figure 2:**
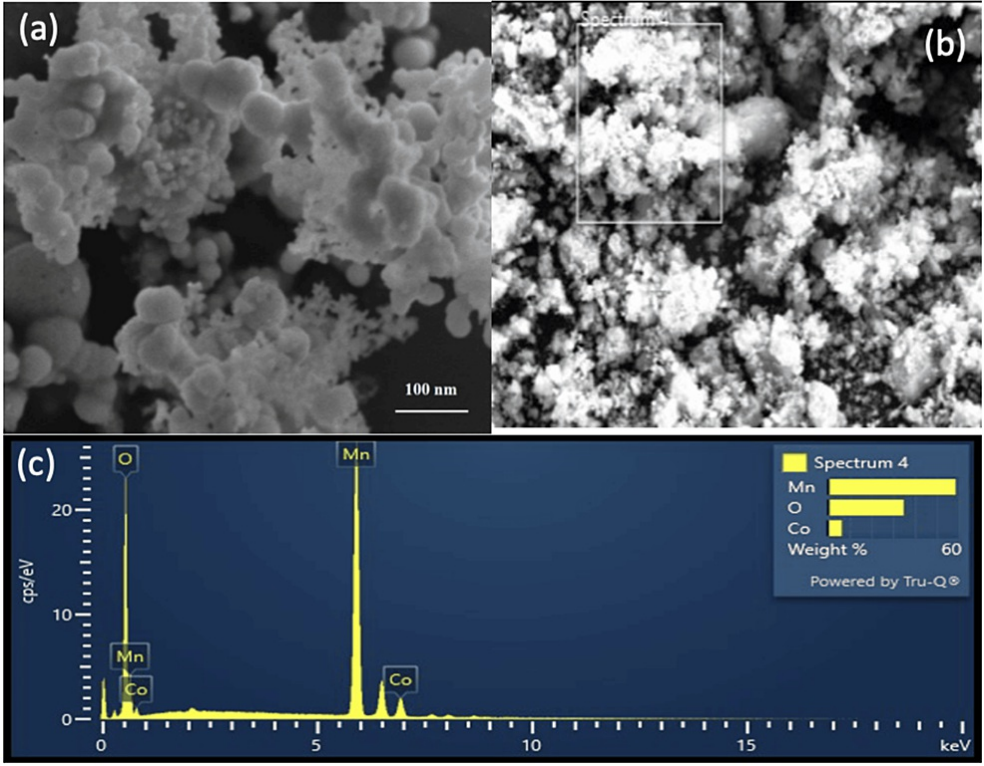
FE-SEM and EDAX analysis of Co-doped MO (a, b) Represent the FE-SEM images of cobalt-doped manganese oxide (Co-doped MO) nanoparticles. (c) Energy-dispersive X-ray spectroscopy of the Co-doped MO. Co, cobalt; MO, manganese oxide; FE-SEM, field emission scanning electron microscopy; EDAX, energy-dispersive X-ray spectroscopy.

The FE-SEM images of the Co-doped MO samples are presented in Figure 2a, 2b. The micrographs reveal intricate details of the sample's surface morphology and microstructure. The images show that the Co-doped MO materials exhibit well-defined spherical structures, with varying degrees of agglomeration depending on the Co doping concentration with the nanoparticle size of 100 nm. EDAX in Figure 2c shows the presence of Co, Mn, and O, which confirms the purity of the synthesized sample (Figure 2).

**Figure 3:**
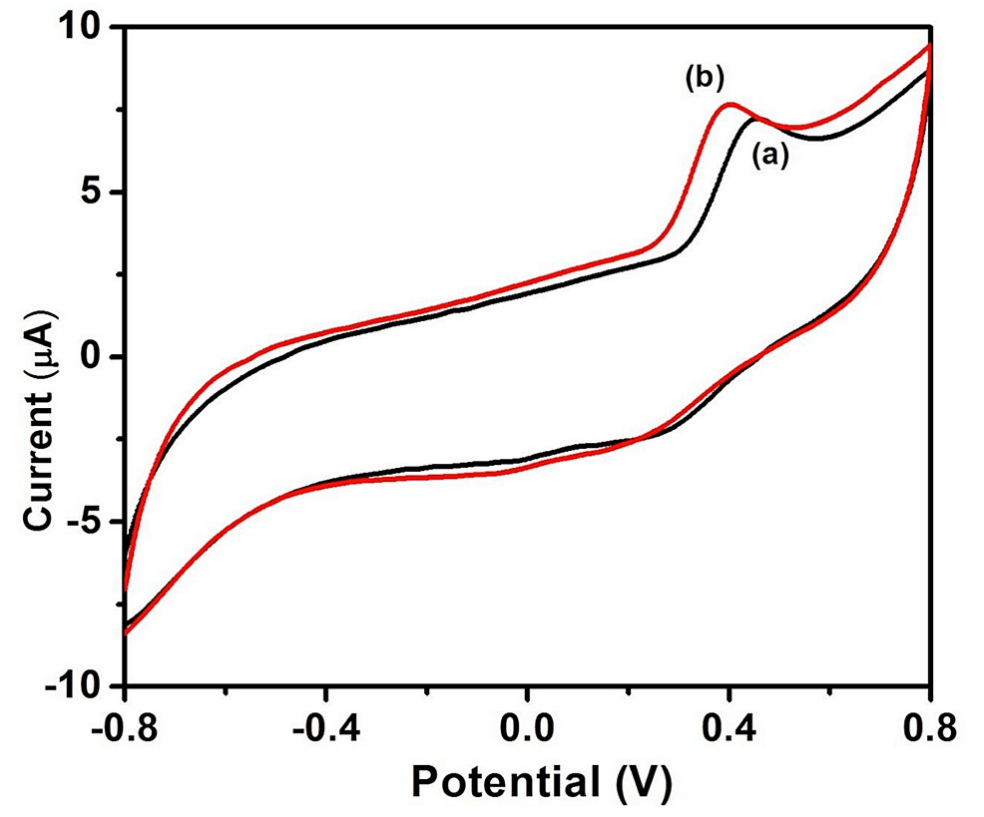
Cyclic voltammetry response of Co-doped MO toward quercetin at 50 mV/s Co, cobalt; MO, manganese oxide.

The study examined the cyclic voltammetric reactions of bare GCE (a) and Co-doped MO/GCE (b) to quercetin in phosphate buffer (Na_2_HPO_4_ + NaH_2_PO_4_, pH 7) at a scan rate of 50 mV (Figure 3).

**Figure 4:**
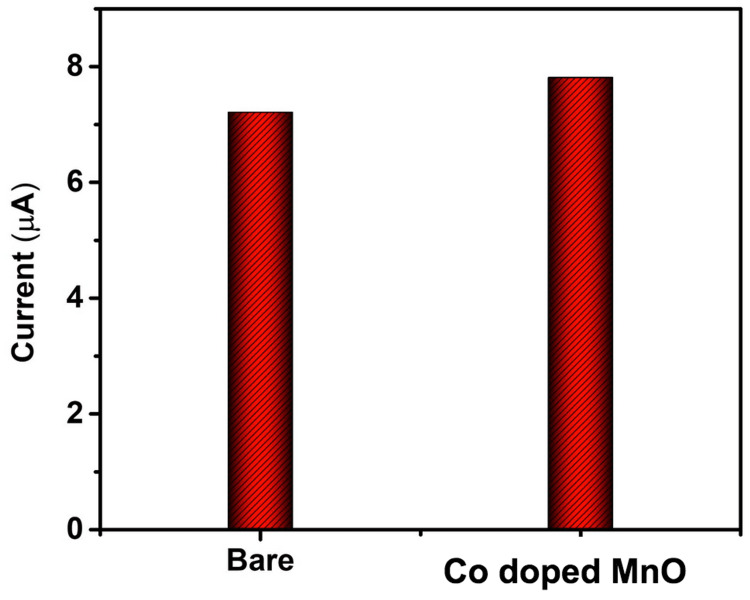
Cyclic voltammetry current response of bare and Co-doped MnO-modified electrode toward quercetin. Co, cobalt; MO, manganese oxide.

The current response of bare and modified electrodes toward quercetin is shown as a bar diagram (Figure 4).

**Figure 5:**
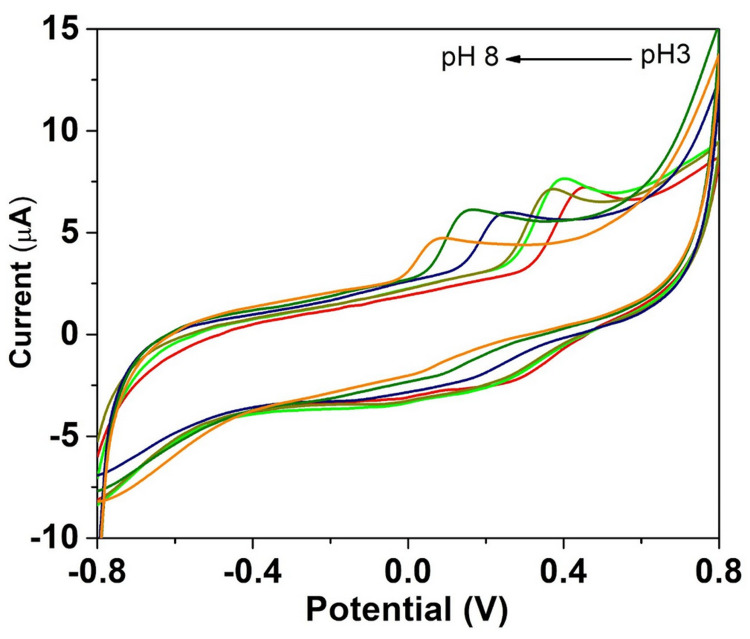
Co-doped MO/GCE cyclic voltammograms at various pH values observed at a scan rate of 50 mV Co, cobalt; MO, manganese oxide; GCE, glassy carbon electrode.

CV was used to assess the impact of pH on the oxidation of 0.1 mM quercetin at the Co-doped MnO/GCE in the pH range of 3-8. Figure 5 displays the resulting cyclic voltammograms.

**Figure 6:**
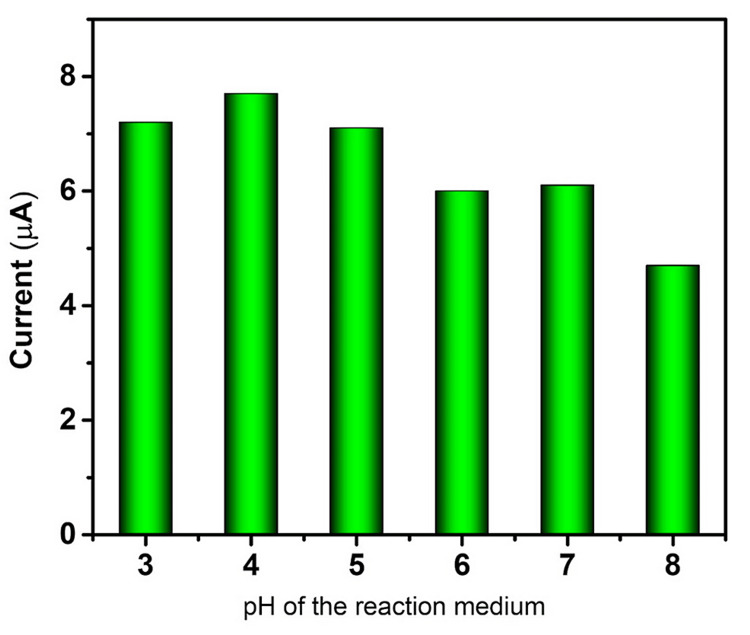
pH vs current response of Co-doped MO toward quercetin Co, cobalt; MO, manganese oxide.

Figure 6 shows the pH-dependent current response of the modified electrode among the modified electrodes; the higher current response was observed at pH 4.

## Discussion

Quercetin could be detected by the electrochemical method. Due to its reversible redox reactions, quercetin is an easy electron donor and acceptor. This characteristic is utilized in electrochemical sensing, where electrodes modified with quercetin promote electron transfer reactions with target analytes. Measurable electrical signals are produced by this interaction, and these signals could be connected to the target molecule's concentration [18]. We also utilized the electrochemical method to identify the quercetin biomolecules. To create quercetin-modified electrodes, researchers frequently immobilize quercetin on electrode surfaces. This alteration improves the electrode's selectivity and sensitivity for particular analytes. The immobilization process could be due to covalent bonding or physical adsorption, which guarantees steady and long-lasting sensor performance. Electrodes that have been treated with quercetin are used in biosensing applications to identify a variety of biomolecules, such as proteins, enzymes, and DNA. The target analytes' identification and quantification are made possible by the electrochemical signals generated during these interactions [19]. We have also utilized modified electrodes in our study. 

Amperometry, voltammetry, impedance spectroscopy, and potentiostatic/galvanostatic measurements have been used in the investigation of Co-MO nanostructures for biomolecule sensing [16-18]. Co doping modulates the electronic band structure of MO and modifies the surface area and surface charge distribution, which influences biomolecule adsorption and electrochemical signal transduction. The synergistic effect between Co and manganese can promote catalytic activity, allowing for enhanced signal amplification and reduced detection limits [20]. In our current study, the topographical structure and elemental makeup of Co-doped MO were investigated using EDAX. With no additional impurities, the outcome validated the purity of the produced nanomaterial. FE-SEM images also provided insights into the surface roughness and porosity of the Co-doped MO samples. The presence of cobalt dopants appears to influence the surface texture, resulting in variations in surface roughness and the formation of nanoporous features. These surface modifications could play a significant role in enhancing the electrochemical performance of the material by providing increased surface area and improved accessibility for biomolecule interactions. 

The excellent sensitivity, low cost, and rapid pace at which electrochemical biosensors operate make them intriguing options for the diagnosis of various viral diseases like influenza, Ebola, and human immunodeficiency virus [21,22]. Especially during the COVID-19 pandemic, biosensors have become popular [23,24]. The future scope is to commercially manufacture electrochemical sensors in compact sizes that could be used at home to detect various diseases including diabetes and arthritis [25]. The potential of electrochemical sensors holds great promise for monitoring antineoplastic medication therapy and aiding in detecting cancer [26].

It is evident from the cyclic voltammograms that quercetin is oxidized at GCE at 478 mV with an anodic peak current of 6.17 µA, and quercetin is detected at Co@MO/GCE at a greater current response of 7.35 µA with a lower peak potential of 342 mV. Karuppusamy et al. suggested that the GCE modified with 6 µL of CdTe/BS-RGO/GCE exhibited greater response when tested under 50 µM QR with 0.1 M PB (pH 7.0) at 50 mV [27]. Muthamizh et al. observed that quercetin was oxidized and also exhibited a higher response with a lower peak potential [28]. The voltammograms reveal the pH-dependent redox behavior of quercetin, with distinct oxidation and reduction peaks shifting in response to changes in pH. These results underline the significance of pH regulation and tuning for the creation of electrochemical sensors that are targeted at certain biomolecules. This confirmed the superiority of Co-doped MO over GCE. The metal ion, smaller crystallite size, and surface hydroxyl group could be the primary causes of the increased electrochemical activity at reduced potential.

Quercetin is a flavonoid polyphenolic compound that has numerous biological effects. It is shown to have anti-inflammatory effects on lung epithelial cells [29] and periodontal fibroblasts [30]. We have shown that our Co-doped MO could detect quercetin at higher accuracy. The future scope will be the incorporation of these Co-doped MnO biosensors into point-of-care diagnostics that will enable chairside detection of various biomarkers in serum, saliva, sputum, and other bodily fluids. It could be synergized with the identification of microbial products, antibiotic sensitivity, and other potential applications. Further in vitro evaluation of cell cultures, animal studies, and clinical trials will help in translating this novel compound into a diagnostic device.

The limitations of our study are mentioned here. Interference from other substances in the sample matrix might affect the electrochemical detection process. Electrochemical detection may be difficult with samples that have complex matrices, including biological fluids or food extracts. Quantification challenges may arise due to matrix components interfering with quercetin's signal. Detecting low amounts of quercetin may not always be possible using electrochemical techniques due to their limited sensitivity, particularly in diluted samples. This constraint can be addressed by applying pre-concentration techniques or more sophisticated electrochemical procedures.

## Conclusions

The incorporation of cobalt dopants into the MO lattice has been shown to induce structural modifications that enhance conductivity, reactivity, and electrochemical performance. The investigation of Co-doped MO as a prospective candidate for increasing biomolecule sensing using electrochemical techniques was the focus of this paper. Through comprehensive XRD and FE-SEM analyses, we gained valuable insights into the structural characteristics and microstructural changes induced by Co doping, shedding light on the potential mechanisms underlying the enhanced sensing capabilities of Co-doped MO. Additionally, the CV responses of Co-doped MO to quercetin clarified the material's redox behavior and pH sensitivity, providing essential knowledge for maximizing sensor performance. This Co-doped MO could be used to design point-of-care diagnostics in the near future.
